# Tension Gastrothorax in a Child With Undiagnosed Congenital Diaphragmatic Hernia: A Case Report and Proposed Emergency Management Algorithm

**DOI:** 10.1155/crpe/3569669

**Published:** 2026-09-29

**Authors:** Kalliopi Straka, Eleni Christakou

**Affiliations:** ^1^ Paediatric Intensive Care Unit, Aghia Sophia Children’s Hospital, Athens, Greece, paidon-agiasofia.gr; ^2^ Medical School, National and Kapodistrian University of Athens, Athens, Greece, uoa.gr

**Keywords:** Bochdalek hernia, congenital diaphragmatic hernia, mediastinal shift, pediatric emergency, respiratory distress, tension gastrothorax

## Abstract

**Background:**

Tension gastrothorax is a rare but life‐threatening pediatric emergency that most commonly results from herniation of the stomach through an unrecognized congenital diaphragmatic defect, followed by progressive intrathoracic gastric distension and compression of intrathoracic structures. Because its clinical presentation often mimics tension pneumothorax, delayed diagnosis, or inappropriate management may rapidly lead to obstructive shock, respiratory failure, and cardiac arrest. Prompt recognition and timely intervention are therefore essential to prevent fatal outcomes.

**Case Presentation:**

We report the case of a previously healthy 3‐year‐old boy who presented with abdominal pain, recurrent vomiting, fever, and rapidly progressive respiratory distress. Shortly after admission, he developed severe respiratory compromise, hemodynamic instability, and altered mental status, raising initial suspicion of tension pneumothorax. However, chest radiography demonstrated a large air–fluid level occupying the left hemithorax with marked mediastinal shift, consistent with tension gastrothorax. Initial nasogastric decompression was unsuccessful, due to distortion of the gastroesophageal junction. Owing to the patient’s critical condition, emergency transthoracic needle decompression followed by drain placement into the distended intrathoracic stomach was performed, resulting in immediate hemodynamic and respiratory improvement. Computed tomography subsequently confirmed a left‐sided Bochdalek congenital diaphragmatic hernia with intrathoracic herniation of the stomach, spleen, small bowel, and part of the colon. Definitive surgical repair was undertaken on the same day after patient’s stabilization. The postoperative course was complicated by acute lung injury and sepsis, but the child recovered fully and remained asymptomatic at follow‐up.

**Conclusion:**

Tension gastrothorax should be considered in the differential diagnosis of any child presenting with sudden respiratory deterioration, unilateral absent breath sounds, and mediastinal shift, especially when preceded by vomiting or other events associated with increased intra‐abdominal pressure. Recognition of the characteristic radiographic findings, prompt gastric decompression, and early multidisciplinary management are critical for preventing life‐threatening cardiorespiratory compromise. This case highlights the importance of maintaining a high index of suspicion for this uncommon but potentially fatal condition in children with previously undiagnosed congenital diaphragmatic hernia.

## 1. Introduction

Tension gastrothorax is a rare but life‐threatening pediatric emergency that results from acute herniation of the stomach into the thoracic cavity through a diaphragmatic defect, followed by progressive intrathoracic gastric distension. The increasing intrathoracic pressure compresses the ipsilateral lung, displaces the mediastinum, impairs venous return and may rapidly lead to obstructive shock, respiratory failure, and cardiac arrest if not promptly recognized and treated [1–3]. Because its clinical and radiographic features closely resemble those of tension pneumothorax, delayed, or inappropriate management may have catastrophic consequences.

In children, tension gastrothorax most commonly occurs as a complication of previously undiagnosed congenital diaphragmatic hernia (CDH), particularly a left‐sided posterolateral (Bochdalek) defect [1, 2, 4, 5]. Although the majority of CDH cases are diagnosed antenatally or during the neonatal period because of severe respiratory compromise, approximately 5%–25% present later in infancy or childhood [2, 5–7]. These late‐presenting cases often remain clinically silent for years or cause only vague, intermittent gastrointestinal or respiratory symptoms, making diagnosis particularly challenging.

Acute deterioration is thought to occur when a precipitating event, such as vomiting, coughing, crying, physical exertion, or any condition associated with increased intra‐abdominal pressure, promotes further herniation, and rapid gastric distension within the thoracic cavity [2, 5, 8]. Progressive enlargement of the intrathoracic stomach may create a functional one‐way valve at the gastroesophageal junction, preventing gastric decompression while allowing continued accumulation of swallowed air and gastric contents. The resulting tension physiology produces rapid mediastinal shift, impaired venous return, reduced cardiac output, and severe ventilation–perfusion mismatch, closely mimicking tension pneumothorax and creating a true pediatric surgical emergency [1–3].

Early diagnosis remains difficult because of the rarity of the condition and its nonspecific presentation. Chest radiography is usually the initial imaging modality and may demonstrate a large intrathoracic air–fluid level, absence of the normal subdiaphragmatic gastric bubble, compression of the ipsilateral lung, and contralateral mediastinal shift. However, these findings may be misinterpreted, particularly in emergency settings, and additional imaging may occasionally be required to confirm the diagnosis and guide definitive surgical management [2, 5, 8].

Herein, we report the case of a previously healthy three‐year‐old boy who developed acute tension gastrothorax secondary to an unrecognized CDH, presenting with rapidly progressive respiratory failure and obstructive shock. In addition to describing the diagnostic and therapeutic challenges encountered during emergency management, we review the current literature and propose a practical evidence‐based emergency management algorithm to facilitate early recognition and timely intervention in this uncommon but potentially fatal condition.

## 2. Case Presentation

### 2.1. Clinical Presentation

A previously healthy 3‐year‐old boy presented to the Emergency Department of our tertiary pediatric hospital with a 10‐h history of abdominal pain, recurrent nonbilious vomiting, malaise, and progressively worsening respiratory distress. According to his caregivers, his oral intake had significantly decreased during the preceding hours, and he had become increasingly lethargic. There was no history of trauma, previous thoracic or abdominal surgery, previous hospital admissions, chronic respiratory disease, or known congenital anomalies. His immunizations were up to date, and his growth and developmental milestones were appropriate for his age. No relevant family history was reported.

On initial assessment, the child appeared acutely ill, febrile (axillary temperature 38.5°C), tachypnoeic, somnolent, and clinically dehydrated. He exhibited grunting respirations and increased work of breathing but remained hemodynamically stable. Given the combination of fever, recurrent vomiting, reduced oral intake, and progressive respiratory symptoms, the initial working diagnosis included acute gastroenteritis with dehydration and a lower respiratory tract infection. Initial management consisted of intravenous fluid resuscitation, antipyretic therapy, baseline laboratory investigations, and close clinical observation while further diagnostic evaluation was being arranged.

Shortly after his admission to the pediatric ward, the patient’s condition deteriorated abruptly. He developed rapidly worsening respiratory distress, increasing oxygen requirements, poor peripheral perfusion, and progressive alteration in mental status, prompting immediate review by the Pediatric Intensive Care Unit (PICU) team and urgent transfer for advanced assessment and resuscitation.

### 2.2. Assessment and Acute Management

Because of the patient’s rapid clinical deterioration following admission to the pediatric ward, he was transferred urgently to the PICU for advanced assessment and resuscitation before further diagnostic investigations could be completed. Given the severity of his clinical deterioration, no imaging studies had been performed before transfer.

Upon arrival in the PICU, the child appeared critically ill and in impending respiratory failure and circulatory compromise. He was markedly tachypnoeic with pronounced subcostal and intercostal retractions, grunting respirations, and worsening lethargy. His heart rate ranged between 190 and 200 beats/min, blood pressure was 100/60 mmHg, and capillary refill time was prolonged, indicating impaired peripheral perfusion despite preserved arterial blood pressure. Oxygen saturation remained between 97% and 99% while receiving supplemental oxygen via face mask at 5 L/min. His chest auscultation revealed markedly reduced air entry over the left hemithorax, while a prominent right precordial pulse suggested significant mediastinal displacement. His Glasgow Coma Scale score was 10/15.

The combination of acute respiratory distress, unilateral absent breath sounds, mediastinal displacement, and evolving hemodynamic compromise initially raised strong clinical suspicion of tension pneumothorax. Nevertheless, because the patient remained sufficiently stable to undergo immediate bedside imaging, urgent chest radiography was obtained before any invasive intervention.

The initial chest radiograph demonstrated a large air–fluid level occupying most of the left hemithorax, marked compression of the ipsilateral lung, and substantial mediastinal shift to the right (Figure 1). In addition, the normal subdiaphragmatic gastric bubble was absent. These findings were highly suggestive of tension gastrothorax rather than tension pneumothorax and, together with the preceding history of recurrent vomiting and rapidly progressive respiratory deterioration, strongly supported the diagnosis of acute intrathoracic gastric distension secondary to an undiagnosed CDH.

**FIGURE 1:**
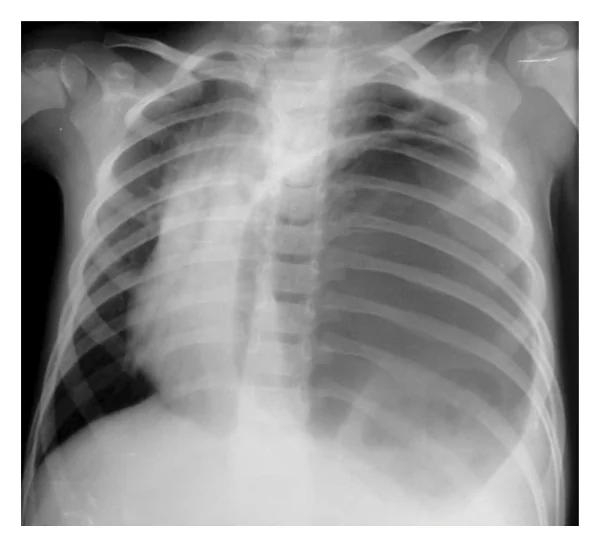
Initial chest radiograph demonstrating a large air–fluid level within the left hemithorax, with marked mediastinal shift to the right and compression of the ipsilateral lung.

Because the child had developed obstructive shock physiology, immediate gastric decompression was considered essential. A large‐bore nasogastric tube was inserted as the initial decompressive measure; however, repeated attempts were unsuccessful, most likely because herniation and torsion of the stomach had resulted in distortion of the gastroesophageal junction. In view of the patient’s rapidly worsening clinical condition and the failure of nasogastric decompression, emergency transthoracic decompression was undertaken by the attending pediatric thoracic surgeon. Needle decompression was first performed, followed by placement of a drain into the distended intrathoracic stomach, resulting in immediate evacuation of air, rapid improvement in respiratory mechanics, restoration of hemodynamic stability, and resolution of the obstructive physiology. A repeat chest radiograph confirmed successful gastric decompression, partial re‐expansion of the left lung, and marked reduction of the mediastinal shift (Figure 2).

**FIGURE 2:**
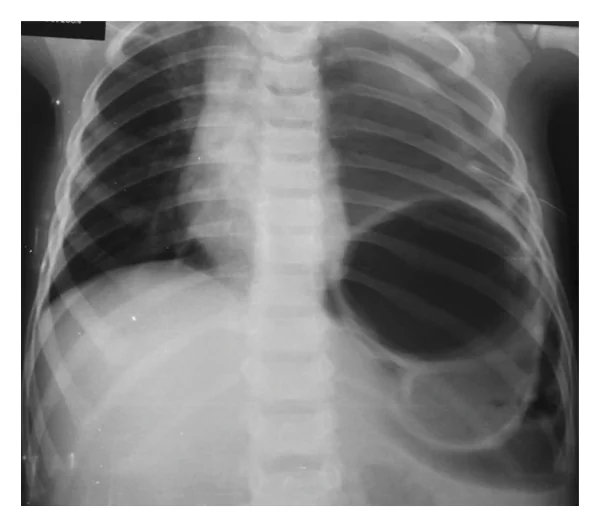
Chest radiograph obtained after emergency decompression and chest drain placement, showing decompression of the intrathoracic stomach and resolution of the mediastinal shift.

### 2.3. Investigations and Diagnostic Approach

Following successful emergency decompression and initial hemodynamic stabilization, further diagnostic evaluation was undertaken to confirm the underlying anatomical abnormality and guide definitive surgical management.

Baseline laboratory investigations demonstrated inflammatory markers consistent with an acute systemic inflammatory response but were otherwise nonspecific. Given the patient’s abrupt clinical deterioration and the characteristic radiographic findings, computed tomography (CT) of the thorax and abdomen was performed after stabilization. His CT confirmed a left‐sided CDH through a posterolateral diaphragmatic defect, with herniation of the stomach, spleen, small bowel, and part of the transverse colon into the left hemithorax. The herniated viscera resulted in marked compression of the left lung and persistent rightward mediastinal displacement, confirming the diagnosis of tension gastrothorax secondary to a previously undiagnosed CDH (Bochdalek type).

The patient’s prenatal history was subsequently reviewed. The pregnancy had been uncomplicated, and routine antenatal ultrasonography, including the anomaly scan, had not identified any congenital abnormalities. There was no history of polyhydramnios or prenatal suspicion of CDH. These findings were consistent with a late‐presenting CDH that had remained clinically silent until this acute event.

Following stabilization, transthoracic echocardiography demonstrated normal cardiac anatomy and function, with no evidence of pulmonary arterial hypertension or associated congenital cardiac abnormalities. These findings were important in excluding additional causes of hemodynamic instability and supported definitive surgical intervention without the need for preoperative cardiopulmonary optimization.

Based on the clinical presentation, characteristic radiographic appearance, CT findings, and intraoperative correlation, the final diagnosis was acute tension gastrothorax secondary to a previously undiagnosed left‐sided CDH.

### 2.4. Therapeutic Intervention and Definitive Management

After having undergone the successful emergency decompression and restoration of hemodynamic stability, definitive surgical repair under general anesthesia was performed on the patient on the same day. Through a laparotomy, a left‐sided posterolateral congenital diaphragmatic defect was identified. The herniated abdominal viscera, including the stomach, spleen, small bowel, and part of the colon, were carefully reduced into the abdominal cavity.

The diaphragmatic defect was subsequently repaired without intraoperative complications. The early postoperative course was complicated by acute lung injury and sepsis, which were considered most likely secondary to pleural contamination following emergency gastric decompression. The patient required invasive mechanical ventilation for 6 days because of persistent respiratory failure and elevated oxygen and ventilatory requirements. During this period, he also developed hemodynamic instability requiring vasoactive support. Broad‐spectrum intravenous antimicrobial therapy consisting of piperacillin/tazobactam, vancomycin, and metronidazole was initiated empirically and subsequently tailored according to microbiological findings. Pleural fluid cultures grew *Pseudomonas aeruginosa*, while cultures obtained from the surgical drain yielded *Serratia marcescens* and *Enterobacter cloacae*. Antimicrobial treatment was continued for a total of 10 days with progressive clinical and laboratory improvement. Inflammatory markers peaked during the early postoperative period, with a maximum C‐reactive protein concentration of 226 mg/L (reference value < 5 mg/L) and a procalcitonin level of 86 ng/mL (reference value < 0.25 ng/mL), before gradually returning towards normal.

Serial chest radiographs demonstrated progressive re‐expansion of the left lung without evidence of recurrent herniation or pleural collection. Transthoracic echocardiography confirmed normal cardiac anatomy and function, with no evidence of pulmonary arterial hypertension. CT of the brain was unremarkable. Following gradual withdrawal of sedation, neurological examination revealed mild generalized hypotonia and transient upper‐limb tremor, both of which resolved completely during recovery without specific intervention.

Nutritional support was initially provided through total parenteral nutrition for 6 days. Enteral feeding was then gradually reintroduced via a nasogastric tube and was well tolerated before transition to full oral feeding. Surgical drains were removed on postoperative day five after satisfactory clinical and radiological improvement.

The patient was successfully extubated on postoperative day six. Respiratory and hemodynamic status improved steadily thereafter, allowing discontinuation of vasoactive support and transfer from the PICU to the pediatric surgical ward after 9 days. He was discharged home 3 days later in good clinical condition.

### 2.5. Follow‐Up and Outcome

The patient’s clinical condition improved progressively throughout his hospitalization, with complete resolution of respiratory distress, normalization of hemodynamic status, and gradual recovery of gastrointestinal function. He was discharged home in good general condition 12 days after admission, tolerating a full oral diet and requiring no respiratory support.

At 1‐month follow‐up, he remained asymptomatic, with no respiratory or gastrointestinal complaints. Physical examination was unremarkable, and follow‐up chest radiography demonstrated complete expansion of the left lung with no evidence of recurrent diaphragmatic herniation or other postoperative complications. His growth and activity were appropriate for age, and no late complications were observed.

## 3. Discussion

### 3.1. Pathophysiology and Late‐Presenting CDH

Tension gastrothorax is a rare but potentially fatal complication of late‐presenting CDH. Unlike uncomplicated CDH, the life‐threatening manifestations of tension gastrothorax are primarily caused by progressive intrathoracic gastric distension rather than the diaphragmatic defect itself. Increasing intrathoracic pressure compromises pulmonary expansion, displaces the mediastinum, impairs venous return, and may rapidly lead to obstructive physiology with cardiorespiratory compromise if prompt decompression is not achieved [1–3].

Late‐presenting CDH accounts for approximately 5%–25% of all CDHs and may remain clinically silent for months or even years before diagnosis [2, 5–8]. Many affected children present with intermittent gastrointestinal or respiratory symptoms, whereas others remain completely asymptomatic until an acute precipitating event results in sudden intrathoracic gastric distension. In the present case, repeated vomiting most likely acted as the triggering event leading to rapid gastric dilatation, mediastinal shift, and severe cardiorespiratory compromise. Similar mechanisms have been described in previously reported pediatric cases of tension gastrothorax associated with late‐presenting CDH [2, 5, 8, 9].

### 3.2. Diagnostic Challenges and Differential Diagnosis

The principal diagnostic challenge in tension gastrothorax is its close clinical resemblance to tension pneumothorax. Both conditions may present with sudden respiratory distress, unilateral absent or markedly reduced breath sounds, tachycardia, hypoxemia, hypotension, mediastinal shift, and signs of obstructive shock. Consequently, tension pneumothorax is frequently considered the initial diagnosis, particularly in children presenting with abrupt cardiorespiratory deterioration. The main differential diagnoses of pediatric emergencies presenting with sudden‐onset respiratory distress, with or without mediastinal shift, are summarized in Table 1, highlighting the distinguishing clinical and radiographic features that may facilitate early diagnosis.

**TABLE 1:** Differential diagnosis of pediatric emergencies presenting with sudden‐onset respiratory distress with and without mediastinal shift.

Differential diagnosis	Typical clinical presentation	Chest X‐ray findings	Key features distinguishing from tension gastrothorax
Tension gastrothorax	Acute respiratory distress, vomiting, abdominal pain, tachycardia, hypotension, altered mental status; signs of obstructive shock	Large air–fluid level in one hemithorax (usually left), mediastinal shift, compressed ipsilateral lung, absent gastric bubble in abdomen	
Tension pneumothorax	Sudden severe respiratory distress, hypotension, tachycardia, unilateral absent breath sounds	Hyperlucent hemithorax with absent lung markings, depressed hemidiaphragm, mediastinal shift	No air–fluid level; no bowel gas pattern; decompression releases air only
Late‐presenting congenital diaphragmatic hernia (nontension)	Recurrent respiratory symptoms, feeding intolerance, vomiting, failure to thrive	Bowel loops or stomach in thorax, variable mediastinal shift	Usually less acute; absence of obstructive shock unless tension develops
Massive pleural effusion/empyema	Fever, respiratory distress, chest pain, sepsis	Homogeneous opacity, meniscus sign, mediastinal shift away from effusion	Fluid density rather than air; no air–fluid gastric level; ultrasound helpful
Pulmonary overinflation/congenital lobar emphysema	Progressive respiratory distress, wheeze, hypoxia	Hyperinflated lobe with mediastinal shift; lung markings present	No air–fluid level; symptoms often progressive rather than sudden
Pulmonary cyst/pneumatocele	Respiratory distress, often post‐infectious	Well‐defined air‐filled cystic lesion	Localised lesion with visible walls; no abdominal organ displacement
Bronchial foreign body aspiration	Sudden cough, choking episode, unilateral wheeze or reduced breath sounds	Air trapping, atelectasis, or initially normal X‐ray	Clear choking history; no mediastinal shift with large air–fluid level
Gastric volvulus (without herniation)	Severe vomiting, epigastric pain, abdominal distension	Enlarged stomach in abdomen	No intrathoracic stomach or mediastinal shift
Mediastinal mass (e.g. lymphoma)	Subacute respiratory distress, cough, weight loss, facial swelling	Widened mediastinum, mass effect	Gradual onset; systemic symptoms; no air–fluid level
Traumatic diaphragmatic rupture	Respiratory distress following blunt or penetrating trauma	Herniation of abdominal contents into thorax	Clear trauma history
Acute severe asthma	Wheeze, prolonged expiration, hypoxia	Hyperinflated lungs, flattened diaphragms	Bilateral findings; no mediastinal shift from mass effect
ARDS/severe pneumonia	Fever, respiratory failure, sepsis	Bilateral or focal infiltrates	No mediastinal shift from mass effect

Chest radiography remains the first‐line imaging modality and often provides the initial diagnostic clue. Typical radiographic findings include a large intrathoracic air–fluid level, absence of the normal subdiaphragmatic gastric bubble, compression of the ipsilateral lung, and contralateral mediastinal shift. Nevertheless, chest radiographs should be interpreted cautiously, as misdiagnosis has been reported in a considerable proportion of published cases, particularly when the herniated stomach is markedly distended or when radiographic appearances are atypical. Therefore, radiographic findings should always be interpreted in conjunction with the clinical presentation and the patient’s hemodynamic status.

Passage of a nasogastric or orogastric tube may serve both diagnostic and therapeutic purposes. When successful, visualization of the tube within the intrathoracic stomach confirms the diagnosis while simultaneously decompressing the stomach and relieving the obstructive physiology. However, failure to advance the tube does not exclude tension gastrothorax, as distortion and torsion of the gastroesophageal junction frequently prevent successful passage, as occurred in our patient.

Additional imaging modalities may be valuable in selected cases. Point‐of‐care thoracic ultrasound has emerged as a useful bedside adjunct in the emergency setting, particularly when the diagnosis remains uncertain. Identification of intrathoracic abdominal viscera, diaphragmatic discontinuity, or bowel peristalsis within the thorax strongly supports CDH, while the presence of normal pleural sliding helps exclude tension pneumothorax. However, ultrasound findings remain operator dependent and should complement rather than replace conventional radiographic assessment.

CT should be reserved for hemodynamically stable patients or performed after successful emergency decompression. It provides definitive confirmation of the diaphragmatic defect, accurately identifies the herniated abdominal viscera, excludes alternative diagnoses, and facilitates operative planning. Importantly, CT should never delay life‐saving decompression in children presenting with obstructive shock.

The present case highlights the importance of maintaining a high index of suspicion for tension gastrothorax in children presenting with sudden respiratory deterioration, particularly when preceded by vomiting or other events associated with increased intra‐abdominal pressure. Careful integration of the clinical history, physical examination, and imaging findings is essential to establish the correct diagnosis promptly and avoid inappropriate interventions that may delay definitive treatment.

### 3.3. Emergency Management and Literature Review

The immediate therapeutic priority in tension gastrothorax is prompt decompression of the distended intrathoracic stomach to reverse the obstructive physiology. Unlike uncomplicated CDH, the life‐threatening component of tension gastrothorax is not the presence of herniated abdominal viscera itself, but the progressive intrathoracic gastric distension causing compression of the lung, mediastinal shift, impaired venous return, and reduced cardiac output. Consequently, emergency decompression should precede definitive surgical repair in patients presenting with hemodynamic instability or impending cardiorespiratory collapse [1, 2, 5, 9, 10].

Current evidence supports nasogastric or orogastric tube insertion as the preferred initial decompressive intervention because it is both diagnostic and therapeutic and avoids the potential complications associated with transthoracic gastric puncture [1, 2, 9, 10]. Successful decompression may rapidly improve respiratory mechanics and hemodynamic status, allowing definitive surgical repair to be performed under more controlled conditions. However, advancement of a gastric tube may be unsuccessful when herniation of the stomach produces marked angulation or torsion of the gastroesophageal junction, preventing passage into the intrathoracic stomach. In such circumstances, repeated attempts should not delay further intervention in a rapidly deteriorating patient.

When gastric tube decompression fails and the child remains hemodynamically unstable, emergency transthoracic needle decompression or placement of a drainage catheter into the distended stomach represents a recognized rescue intervention that may be lifesaving [1, 5, 9, 10]. Although this approach carries inherent risks, including gastric perforation, pleural contamination, and postoperative infection, these potential complications must be weighed against the immediate risk of progressive obstructive shock and cardiac arrest. In our patient, failure of nasogastric decompression, together with rapidly worsening cardiorespiratory compromise, necessitated emergency transthoracic decompression. The immediate hemodynamic and respiratory improvement observed following decompression supports the appropriateness of this strategy under the prevailing clinical circumstances.

Following physiological stabilization, definitive surgical repair should be performed without unnecessary delay. Laparotomy remains the preferred operative approach in the emergency setting because it allows safe reduction of the herniated abdominal viscera, assessment of visceral viability, correction of associated gastric volvulus when present, and repair of the diaphragmatic defect through a single abdominal incision. In contrast, proceeding directly to laparotomy in a child with ongoing obstructive shock, before successful gastric decompression, may increase the risk associated with induction of anesthesia and further hemodynamic deterioration. Therefore, stabilization through gastric decompression before definitive surgery represents the most widely accepted management strategy in critically ill children with tension gastrothorax.

Comparison with previously published pediatric cases demonstrates a remarkably consistent pattern of presentation. As in our patient, most children are previously healthy and present with abrupt respiratory deterioration following vomiting or other events associated with increased intra‐abdominal pressure, leading to an initial diagnosis of tension pneumothorax [2, 5, 8, 9]. Our case additionally illustrates an important but less frequently emphasized aspect of emergency management: although transthoracic decompression may be associated with postoperative infectious complications, it can be lifesaving when conventional gastric tube decompression is unsuccessful. This highlights the importance of individualizing management according to the child’s physiological status while prioritizing immediate reversal of life‐threatening obstructive shock [11–13] .

### 3.4. Clinical Implications, Strengths, and Limitations

The present report also underscores the importance of a structured multidisciplinary approach involving emergency physicians, intensivists, radiologists, anesthesiologists, and pediatric surgeons. Rapid interpretation of imaging findings, timely gastric decompression, and prompt definitive surgical repair were fundamental to the favorable outcome observed in our patient. We believe that the proposed evidence‐based management algorithm may facilitate earlier recognition and support clinical decision‐making in this uncommon but life‐threatening pediatric emergency.

The principal strength of this report lies in the comprehensive description of the diagnostic process, emergency stabilization, definitive surgical management, postoperative intensive care, and clinical follow‐up. Furthermore, by integrating the available literature with our clinical experience, we propose a practical management algorithm that may assist clinicians faced with this rare presentation.

The limitations of this report are those inherent to a single‐case study. Management decisions were based on the patient’s clinical condition and available institutional expertise and therefore cannot be generalized to all cases. Nevertheless, given the rarity of tension gastrothorax in children, carefully documented case reports remain valuable for improving awareness, facilitating earlier diagnosis, and guiding future clinical practice.

### 3.5. Proposed Emergency Management Algorithm for Suspected Tension Gastrothorax in Children

Figure 3 summarizes a practical, evidence‐informed approach to the emergency assessment and management of children with suspected tension gastrothorax. Initial management prioritizes airway, breathing, and circulation (ABC), followed by rapid recognition of characteristic clinical and radiographic findings. Nasogastric or orogastric tube decompression should be attempted as the first‐line intervention whenever feasible. In patients with persistent hemodynamic instability or unsuccessful gastric tube decompression, emergency transthoracic decompression should not be delayed. CT is recommended after physiological stabilization to confirm the diagnosis and facilitate operative planning. Definitive surgical repair should follow stabilization, with subsequent postoperative intensive care and clinical follow‐up. The algorithm was developed by integrating the clinical course of the present case with evidence from the published literature and is intended as an educational aid to support, rather than replace, individualized clinical judgement.

**FIGURE 3:**
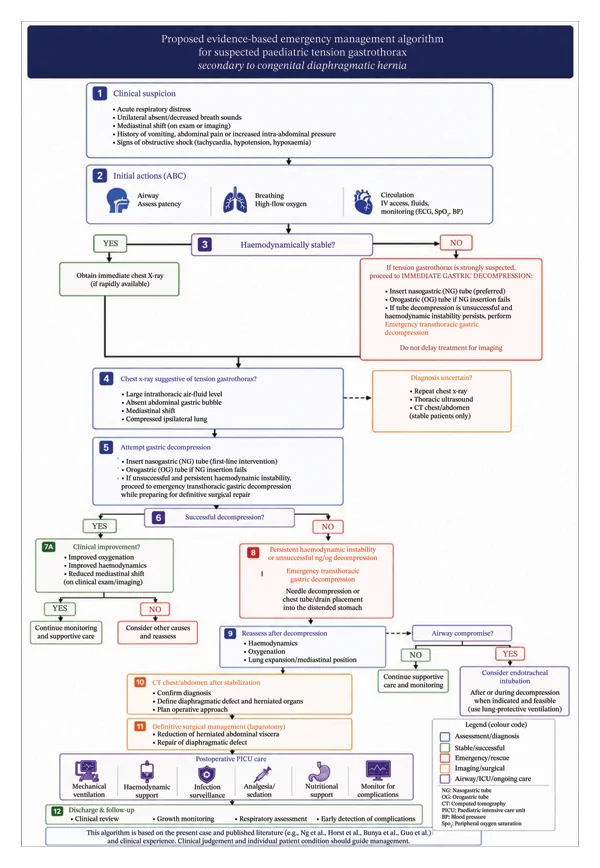
Proposed evidence‐based emergency management algorithm for suspected tension gastrothorax in children with late‐presenting congenital diaphragmatic hernia.

The proposed management algorithm was developed by integrating the clinical course of the present case with recommendations from previously published pediatric series and contemporary emergency management strategies, particularly those described by Ng et al., Bunya et al., Horst et al., and Guo et al. It is intended as a practical educational tool to facilitate early recognition and timely management of this uncommon but life‐threatening condition rather than as a substitute for individualized clinical judgement [14, 15].

## 4. Conclusions

Tension gastrothorax is a rare but life‐threatening complication of late‐presenting CDH that requires prompt recognition and immediate intervention. Because its clinical presentation closely mimics tension pneumothorax, a high index of suspicion is essential in children presenting with sudden respiratory deterioration, unilateral absent breath sounds, and mediastinal shift, particularly when preceded by vomiting or other events associated with increased intra‐abdominal pressure.

This case highlights the importance of careful radiographic interpretation, timely gastric decompression, and coordinated multidisciplinary management in preventing progressive life‐threatening cardiorespiratory collapse. Definitive surgical repair following physiological stabilization can result in excellent clinical outcomes, even in critically ill patients.

Although uncommon, tension gastrothorax should remain an important differential diagnosis in pediatric emergency and critical care practice. Increased awareness of this entity and a structured, evidence‐based approach to diagnosis and management may facilitate earlier recognition, reduce diagnostic errors, and improve patient outcomes.

### 4.1. Learning Points

• Tension gastrothorax should be considered in any child presenting with sudden respiratory distress, unilateral absent breath sounds, and mediastinal shift, as its clinical presentation may closely mimic tension pneumothorax.
• Careful interpretation of chest radiography is essential. A large intrathoracic air–fluid level, absence of the normal gastric bubble, and mediastinal shift should prompt suspicion of tension gastrothorax, while CT may confirm the diagnosis after initial stabilization.
• Nasogastric or orogastric decompression is the preferred initial intervention. When this is unsuccessful and the patient remains hemodynamically unstable, emergency transthoracic decompression may be lifesaving and should not be delayed until definitive surgical management can be performed.
• Successful management requires rapid multidisciplinary collaboration among emergency physicians, intensivists, radiologists, anesthesiologists, and pediatric surgeons to achieve timely diagnosis, physiological stabilization, and definitive surgical repair.

## Author Contributions

The authors contributed equally to the conception, drafting, and revision.

## Funding

No funding was received for the preparation of this manuscript.

## Disclosure

All authors approved the final version of the manuscript. The authors received no financial support, technical assistance or other external contribution in the preparation of this case report.

## Ethics Statement

The authors declare that the research presented in this manuscript adheres to the ethical principles outlined by the Scientific Committee of Aghia Sophia Children’s Hospital. All procedures involving human participants were conducted in accordance with the ethical standards of the National and Kapodistrian University of Athens and with the 1964 Declaration of Helsinki and its later amendments.

## Consent

Written informed consent was obtained from the patient’s parents for publication of this case report and any accompanying images. A copy of the written consent is available for review by the Editor‐in‐Chief of this journal.

## Conflicts of Interest

The authors declare no conflicts of interest.

## Supporting Information

Additional supporting information can be found online in the Supporting Information section.

## Supporting information


**Supporting Information** CARE checklist.

## Data Availability

The data supporting the findings of this study are available from the corresponding author upon reasonable request.

## References

[1] Ng J. , Rex D. , Sudhakaran N. , Okoye B. , and Mukhtar Z. , Tension Gastrothorax in Children: Introducing a Management Algorithm, Journal of Pediatric Surgery. (2013) 48, 1613–1617, 10.1016/j.jpedsurg.2013.05.066. 23895982

[2] Horst M. , Sacher P. , Molz G. , Willi U. V. , and Meuli M. , Tension Gastrothorax, Journal of Pediatric Surgery. (2005) 40, no. 9, 1500–1504, 10.1016/j.jpedsurg.2005.05.079. 16150360

[3] El Hajj , McCabe M. , Ceppa D. K. , Fridell J. A. , and Sherman S. , Life-Threatening Nontraumatic Tension Gastrothorax, Clinical Case Reports. (2018) 6, 1902–1905, 10.1002/ccr3.1468. PMC5930186 29744098

[4] Fuller G. , Cacala S. , and Oosthuizen G. , Tension Gastrothorax-Colothorax Secondary to Traumatic Diaphragmatic Hernia, Pediatric Emergency Care. (2010) 26, no. 4, 299–301, 10.1097/pec.0b013e3181d6db22. 20386417

[5] Næss P. A. , Wiborg J. , Kjellevold K. , and Gaarder C. , Tension Gastrothorax: Acute Life-Threatening Manifestation of Late-Onset Congenital Diaphragmatic Hernia in Children, Scandinavian Journal of Trauma, Resuscitation and Emergency Medicine. (2015) 23, no. 1, 10.1186/s13049-015-0129-8. PMC4477604 26104782

[6] Wenstrom K. D. , Wiener C. P. , and Hanson J. W. , A Five-Year Statewide Experience With Congenital Diaphragmatic Hernia, American Journal of Obstetrics and Gynecology. (1991) 165, no. 4 Pt 1, 838–842, 10.1016/0002-9378(91)90425-q. 1951540

[7] Langham M. R. , Kays D. W. , Ledbetter D. J. , Frentzen B. , Sanford L. L. , and Richards D. S. , Congenital Diaphragmatic Hernia: Epidemiology and Outcome, Clinics in Perinatology. (1996) 23, no. 4, 671–688. 8982563

[8] Zedan M. , El-Ghazaly M. , Fouda A. , and El-Bayoumi M. , Tension Gastrothorax: A Case Report and Review of the Literature, Journal of Pediatric Surgery. (2008) 43, no. 4, 740–743, 10.1016/j.jpedsurg.2007.10.072. 18405725

[9] Guo R. , Zhang L. , Zhang S. et al., Case Report: Emergency Treatment of Late-Presenting Congenital Diaphragmatic Hernia With Tension Gastrothorax in Three Children, Frontiers in Pediatrics. (2023) 11, 10.3389/fped.2023.1115101. PMC9929136 36816375

[10] Bunya N. , Sawamoto K. , Uemura S. et al., How to Manage Tension Gastrothorax: A Case Report of Tension Gastrothorax With Multiple Trauma due to Traumatic Diaphragmatic Rupture, International Journal of Emergency Medicine. (2017) 10, no. 1, 10.1186/s12245-017-0131-1. PMC5267754 28127711

[11] Ordog G. J. , Wasserberger J. , and Balasubramanian S. , Tension Gastrothorax Complicating Post-Traumatic Rupture of the Diaphragm, American Journal of Emergency Medicine. (1984) 2, no. 3, 219–221, 10.1016/0735-6757(84)90008-1. 6518015

[12] Houas Y. , Oueslati H. , Sghairoun N. , Guitouni A. , and Jouini R. , Tension Gastrothorax in Late Diagnosis of Congenital Diaphragmatic Hernia, Radiology Case Reports. (2025) 20, no. 12, 6203–6206, 10.1016/j.radcr.2025.08.094. PMC12506496 41070290

[13] Shinjo T. , The Need to Suspect Tension Gastrothorax as a Differential Diagnosis of Tension Pneumothorax, International Journal of Surgery Case Reports. (2024) . 10.1016/j.ijscr.2024.109612 PMC11004640 38581944

[14] García Cruz E. J. , López Jiménez A. , Pastrana Rosas J. M. et al., Bochdalek Hernia: Pathophysiology, Clinical Presentation and Management, International Journal of Medical Science and Clinical Research. (2023) .

[15] Koh W. H. , Late-Presenting Congenital Diaphragmatic Hernia: Diagnostic Challenges, Medicine. (2024) .

