# Supplementary material for: Tension Gastrothorax in a Child With Undiagnosed Congenital Diaphragmatic Hernia: A Case Report and Proposed Emergency Management Algorithm
Source: Case Rep Pediatr. 2026 Sep 29;2026:3569669. doi: 10.1155/crpe/3569669 (PMC13620827; doi:10.1155/crpe/3569669)
Supplement: Supplementary file 1 [file CRPE-2026-3569669-s001.docx]

CARE Checklist of information to include when writing a case report

**Topic	Item	Checklist item description	Reported on Line**

**Title	1**	The diagnosis or intervention of primary focus followed by the words “case report” . . . . . . . . . . . . . . . . . .

**Key Words	2**	2 to 5 key words that identify diagnoses or interventions in this case report, including "case report"

**Abstract**

**(no references)**

**3a**	Introduction: What is unique about this case and what does it add to the scientific literature?

**3b**	Main symptoms and/or important clinical findings . . . . . . . . . . . . . . . . . . . . . . . . . . . . . . . . . . . . . . . . . . . . . . . . . . .

**3c**	The main diagnoses, therapeutic interventions, and outcomes

**3d**	Conclusion—What is the main “take-away” lesson(s) from this case?

**Introduction	4**	One or two paragraphs summarizing why this case is unique (**may include** reference**s**)

**Patient Information	5a**	De-identified patient specific information

**5b**	Primary concerns and symptoms of the patient

**5c**	Medical, family, and psycho-social history including relevant genetic information

**5d**	Relevant past interventions with outcomes

**Clinical Findings**

**Timeline**

**Diagnostic Assessment**

**Therapeutic Intervention**

**Follow-up and Outcomes**

6 Describe significant physical examination (PE) and important clinical findings
7 Historical and current information from this episode of care organized as a timeline

**8a**	Diagnostic testing (such as PE, laboratory testing, imaging, surveys).

**8b**	Diagnostic challenges (such as access to testing, financial, or cultural)

**8c**	Diagnosis (including other diagnoses considered)

**8d**	Prognosis (such as staging in oncology) where applicable

**9a**	Types of therapeutic intervention (such as pharmacologic, surgical, preventive, self-care) . . . . . . . . . . . . . . . . .

**9b**	Administration of therapeutic intervention (such as dosage, strength, duration)

**9c**	Changes in therapeutic intervention (with rationale)

**10a**	Clinician and patient-assessed outcomes (if available)

**10b**	Important follow-up diagnostic and other test results

**10c**	Intervention adherence and tolerability (How was this assessed?)

**10d**	Adverse and unanticipated events

**Discussion	11a**	A scientific discussion of the strengths AND limitations associated with this case report

**11b**	Discussion of the relevant medical literature **with references**

**11c**	The scientific rationale for any conclusions (including assessment of possible causes)

**11d**	The primary “take-away” lessons of this case report (without references) in a one paragraph conclusion

**Patient Perspective	12**	The patient should share their perspective in one to two paragraphs on the treatment(s) they received . . . .

**Informed Consent	13**	Did the patient give informed consent? Please provide if requested . . . . . . . . . . . . . . . . . . . . . . . . . . . . . . . . . . . . . .	**Yes** **No**
